# Temperaments

**Published:** 1900-12-15

**Authors:** Gustavus North

**Affiliations:** Cedar Rapids, Iowa


### TEMPERAMENTS.

BY GUSTAVUS NORTH, A.M., D.D.S., CEDAR RAPIDS, IOWA.

Read before the Southwestern Michigan Dental Association.

The subject of temperaments presents itself to my mind as one of great importance. There has been very little written upon this subject, and practically no attention paid to its interesting peculiarities. I feel very deeply interested in this subject, and it certainly should be of equal interest to all in the profession, especially those interested in artistic dentistry.

Do you understand temperaments? Can you read the different types of temperaments, and describe the teeth of each? If you can not do this, then you should consider the matter. It is a study very easy to comprehend and very interesting in all its details. As I can not be with you at this meeting to bring this subject before you more fully, I will only write a short paper, and hope I may bring out some points worthy of discussion. Some time in the future I want to publish a little work on temperaments, fully illustrated, to assist in the practice of artistic dentistry. I believe I can in two or three lectures teach a class of students so that they will be able to distinguish temperaments.

There are four basal temperaments and twelve subtemperaments, but in this paper I will speak only of the basal, as follows:

*Bilious temperament.*—Spare, angular development; characterized by a preponderance of bile; dark, sallow complexion; hair and eyes dark; lips purple; teeth of a yellowish color, rather narrow at the neck, and long in proportion to the width; articulation firm and close.

*Sanguine temperament.*—Generally well developed; fair complexion; light brown hair; blue or gray eyes; full red lips; teeth cream color, average in size, about the same at the neck as at the cutting edge, with rounded cusps; gums round and full; articulation moderately firm; jaw inclined to rotate in masticating.

*Lymphatic temperament.*—Bulky development, looseness of tissue tending toward feebleness; skin pallid; hair either light or dark, thin and straight, and of a soft and matted nature; eyes light or dark; clear mind; circulation feeble; pulse weak; teeth dark, rather clouded in appearance, large and broad, with thick and rounded cusps; articulation loose and flat.

*Nervous temperament.*—Body slender, generally well formed; light complexion; muscles small and yielding; nervous, quick and excitable; hair light or dark; teeth long, decidedly narrower at the neck than at the cutting edge, pearl blue or gray, with long bite, cusps prominent; articulation close and penetrating.

If temperaments are understood, it is an easy matter to select teeth to harmonize with the person, and without any knowledge of the person we should be able to describe their temperament from an extracted tooth. If a tooth is long, decidedly narrower at the neck than at the cutting edge, with a long bite, pearl blue or gray in color, articulation close and penetrating, it would certainly be from a person of nervous temperament; and so on, we might pass through the different types of peculiarities.

If temperaments are not understood, it would be very difficult to practice artistic dentistry.

An occulist in supplying an artificial eye selects size, shape and color to harmonize with the person. For him to use a blue eye where a dark or black one was required would be no more out of place than to use nervous temperament teeth for a person of sanguine temperament; or if a rose bush was stripped of its roses and we would endeavor to supply it with sunflowers, they would harmonize as well as many cases that are brought to my notice.

In artistic dentistry the aim should be to supply nature, and this can only be done by a careful study of temperaments.

